# Functional Impact of Protein–RNA Variation in Clinical Cancer Analyses

**DOI:** 10.1016/j.mcpro.2023.100587

**Published:** 2023-06-07

**Authors:** Gali Arad, Tamar Geiger

**Affiliations:** 1 Protai Bio, Givatayim, Israel; 2 Department of Molecular Cell Biology, Weizmann Institute of Science, Rehovot, Israel

**Keywords:** cancer, clinical proteomics, proteogenomics, drug targets, biomarkers

## Abstract

Comprehensive molecular characterization of tumors aims to uncover cancer vulnerabilities, drug resistance mechanisms, and biomarkers. Identification of cancer drivers was suggested as the basis for patient-tailored therapy, and transcriptomic analyses were proposed to reveal the phenotypic outcome of cancer mutations. With the maturation of the proteomic field, studies of protein–RNA discrepancies suggested that RNA analyses are insufficient to predict cellular functions. In this article we discuss the importance of direct mRNA–protein comparisons in clinical cancer studies. We make use of the large amount of data generated by the Clinical Proteomic Tumor Analysis Consortium, which includes protein and mRNA expression analyses from the exact same samples. Analysis of protein–RNA correlations showed marked differences among cancer types, and highlighted the protein–RNA similarities and discrepancies among functional pathways and drug targets. Additionally, unsupervised clustering of the data based on protein or RNA showed substantial differences in tumor classification and the cellular processes that differentiate between clusters. These analyses show the difficulty to predict protein levels from mRNAs, and the critical role of protein analyses for phenotypic tumor characterization.

## Highlights

• Deep proteogenomics show average protein–RNA correlation of 0.4 to 0.6 across samples.
• Lung cancers present highest protein–RNA correlations and ovarian cancer the lowest.
• There is a major variance in protein–RNA correlations among functional pathways.
• Driver amplification events retain high RNA–protein correlations compared to mutations.
• Analyses of protein/RNA result in distinct tumor classification in most cancer types.

## Graphical Abstract

**Figure:**
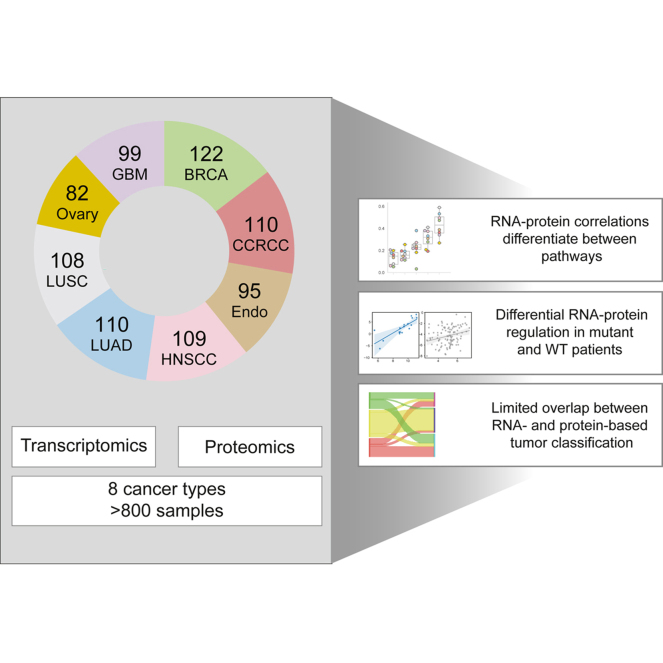

## In Brief

Proteogenomic studies of clinical cancer samples highlight broad protein–RNA discrepancies. Using Clinical Proteomic Tumor Analysis Consortium (CPTAC) protein and mRNA data on eight indications, we show the impact of low RNA–protein correlations on key biological processes, signaling pathways, and drug targets. We demonstrate that low RNA–protein correlations lead to distinct cancer classification, suggesting that protein-based subtyping may unravel distinct clinical features not seen upon RNA measurements. These analyses show the critical role of protein analyses for phenotypic tumor characterization.

Unbiased OMICS approaches revolutionized the way we study cancer. From studies of individual proteins and pathways, we moved to analyzing whole genomes (1), transcriptomes (2), proteomes, and their modifications (3). Large datasets of thousands of genes, transcripts or proteins, across hundreds of clinical cancer samples are then used, in an unbiased manner, to unravel novel cancer biomarkers and drug targets. The literature is dominated by transcriptomic studies due to their high coverage and the broad availability of sequencing facilities. Nevertheless, large efforts are made to add the proteomic layer, thereby providing more functional information about tumor behavior and the downstream impact of mutations (3, 4, 5, 6). Despite the accumulation of high-quality proteomic data in clinical cancer research, the translational contribution of the findings derived from the proteomic layer beyond those derived from RNA sequencing (RNAseq) data still requires systematic investigation.

Multiple studies of protein–RNA correlations in controlled *in vitro* systems showed an overall protein–RNA correlation of ∼0.6 within samples (7, 8, 9). However, depending on the cellular perturbation, the contribution of distinct regulatory mechanisms may vary. For example, analysis of LPS stimulation showed dominant transcriptional control (10), while analysis of protein misfolding stress showed more predominant regulation at the protein level (11). Additional studies defined the mismatch between transcripts and proteins and examined the underlying biological regulation and potential technical variance (12, 13). The regulatory mechanisms that impact protein–RNA correlations have been thoroughly discussed elsewhere (14). In the context of cancer, several studies examined the impact of aneuploidy on RNA and protein expression levels (13, 15, 16, 17). They showed that DNA copy number alterations are mostly compensated at the protein level thereby reducing overall RNA–protein correlations (13). The most characterized examples are protein complexes (*e.g.* ribosomes, spliceosomes) and mitochondrial proteins, which are known to be primarily controlled at the protein level. However, other pathways and processes are strongly regulated at the RNA level, resulting in high protein–RNA correlations (13). Here, we wish to focus on the relevance of protein–RNA discrepancies to clinical cancer research, from basic functional analyses to drug target discovery, tumor classification, and patient stratification.

With improved proteomic technologies and analytical throughput, proteomics-derived insights and their translational significance have increased as well. High-precision mass spectrometry techniques have dramatically improved in the last decade, in terms of coverage, throughput, and cost—allowing the richness of the proteome to be unlocked. As a result, studies analyzing as little as a few dozens of tumor samples were able to challenge RNA-based classification and identify novel markers and drug targets that cannot be found by RNAseq analyses (18, 19, 20, 21, 22). The Clinical Proteomic Tumor Analysis Consortium (CPTAC) has been leading proteogenomic analyses in the last decade (23, 24, 25, 26, 27, 28, 29, 30, 31, 32, 33), integrating genomic sequencing, RNAseq, proteomics, phosphoproteomics, and more (3). Each of these studies included several protein–RNA comparisons (28, 29, 30, 31, 34, 35, 36, 37). The fact that RNAseq and proteomics were done on the exact same tissues eliminated biological variation among the analyses, and enabled accurate determination of the impact and relevance of each expression layer. Here we wish to take advantage of the large number of published clinical cancer proteogenomic studies to systematically investigate RNA–protein correlations in clinical cancer samples and their impact on basic as well as translational research and drug discovery.

## Results

### Protein–RNA Correlations Across Tumor Samples

**Fig. 1:**
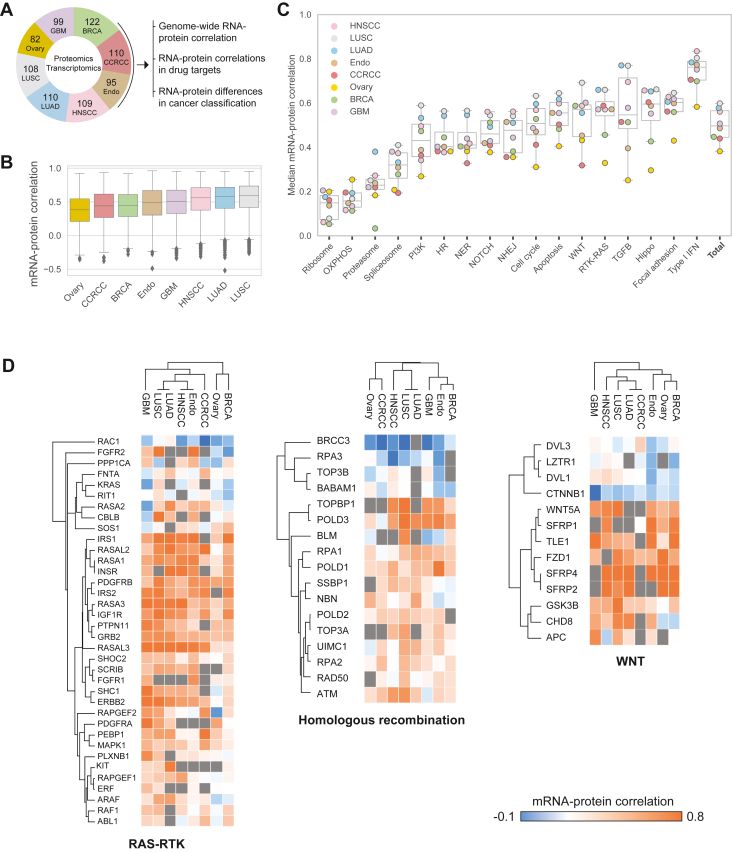
**Protein-RNA correlations across cancer types.***A*, outline of the analysis in this study. Transcriptomic and proteomic data were obtained from eight CPTAC studies spanning eight different cancer types. The number of patients and proteins used in each analysis are included in Supplemental Table S1. *B*, boxplots show the gene-wise protein-RNA Spearman rank correlations in eight cancer types. *C*, separation of the proteomic data to functional pathways, as annotated by GO and KEGG, shows marked differences in protein–RNA correlations within different pathways. Ribosomes, proteasomes, and oxidative phosphorylation show the lowest correlations, and type I interferon (IFN) pathway shows the highest protein–RNA correlations. *D*, hierarchical clustering of protein-RNA correlations of selected pathways shows high variation among pathway components, between different proteins and between tumor types. HR, homologous recombination; NER, nucleotide excision repair; NHEJ, non-homologous end joining; OXPHOS, oxidative phosphorylation; RTK, receptor tyrosine kinase.

We utilized publicly available proteomic and transcriptomic data from CPTAC to analyze multiple specimens in each indication/cancer type and assess the gene-wise correlations across samples (Fig. 1*A* and Supplemental Table S1 for sample numbers). The average Spearman rank correlation ranged from 0.4 to 0.6 in different tumor types, with ovarian cancer and lung squamous cell carcinoma (LUSC) having the lowest and highest average correlations, respectively (Fig. 1*B* and Supplemental Table S2). Interestingly, all tumors presented similarly wide distributions of protein/RNA correlations, ranging from −0.4 to 0.9. Next, we asked whether cancer related biological processes and functions tend to have systematically low or high RNA–protein correlations. In agreement with the individual CPTAC studies, all tumor types present very low protein–RNA correlation of ribosomal proteins, oxidative phosphorylation (OXPHOS) and proteasomes (Fig. 1*C*). These results agree with previous analyses that showed low correlations of large protein complexes, because of reduced stability of unassembled protein subunits (13, 38, 39). In contrast, a well-characterized transcriptionally regulated pathway is the interferon-signaling pathway, which shows the highest protein–RNA correlation (13). Investigation of selected cancer-related pathways (40) showed medium correlations of 0.4 to 0.5 for most pathways, including the PI3K pathway, DNA damage repair (HR and NER, NHEJ), Notch pathway, Wnt pathway, etc. In agreement with the global protein-RNA analysis, lung cancers (squamous cell carcinoma, LUSC and adenocarcinoma, LUAD) and head and neck cancer (HNSCC) presented the highest protein–RNA correlations for most individual pathways, while ovarian cancer and renal cancer (CCRCC) showed the lowest ones. Focusing on the proteins of three of these pathways, we found a large range of RNA-protein correlation within each pathway (Fig. 1*D*). One such classical example is β-catenin (CTNNB1), which is known to be regulated at the protein level by the ubiquitin proteasome system (41). However, in addition to β-catenin, these analyses highlight multiple proteins with consistently low protein-RNA correlations, such as BABAM1, BRCC3, and RAC1. Furthermore, this analysis shows that many of the proteins vary across tumor types. For example, the fibroblast growth factor receptor FGFR2 protein highly correlates with RNA in endometrial cancer (Endo) and LUSC, but not in breast cancer (BRCA) and ovarian cancer.

The mechanisms that control protein and RNA regulatory levels have been thoroughly studied mainly in microorganisms, but they are still not fully elucidated in these complex bulk tumor tissues. Proposed mechanisms include protein subcellular localization, transcriptional negative or positive feedback, chromosomal loci, and more (13, 42). These mechanisms can potentially explain some of the discrepancies between RNA and protein expression. At the cellular levels, proteins are balanced between highly efficient protein production to a more seemingly "wasteful" protein control, which has been proposed to increase cellular robustness in dynamic microenvironments. While the former is reflected in high RNA–protein correlation, the latter is reflected in low correlation (42). The large variance among pathways and tumor types suggests that regulation of protein levels is highly tissue-specific, and therefore, any prediction of protein levels from the RNA levels would have to account for the background activity of multiple regulatory mechanisms in the context of tissue-type and functional state.

**Fig. 2:**
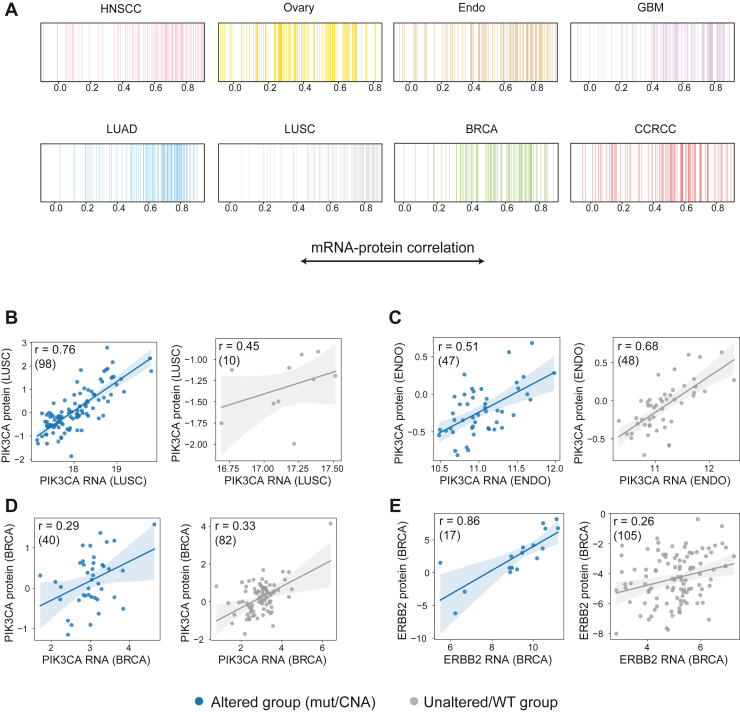
**Protein-RNA associations among cancer therapeutic drugs.***A*, barcode plots show high variation of correlations in all cancer types, including proteins with −0.1 to 0.9 correlation. *B–E*, scatter plots describing the RNA–protein correlations in PIK3CA-altered patients compared to PIK3CA-wt patients in LUSC (*B*), Endo (*C*) and BRCA (*D*); and in ERBB2-altered patients compared to ERBB2-wt patients in BRCA (*E*). In each plot, *blue* represents the altered group and *gray* represents the wt group. Numbers in the *upper left corner* are Spearman correlation followed by number of samples in brackets.

Focusing on the correlations of clinically-relevant proteins, we extracted a list of cancer-relevant drug targets by overlapping FDA-approved drug targets and cancer-related genes curated by the Human Protein Atlas. In agreement with the previous analyses, the average protein–RNA correlations of drug targets ranged from −0.1 to 0.9 in the different tumor types (Fig. 2*A*). We then focused on two key signaling components, PI3K and ErbB2. Both genes are drug targets and frequently altered in cancer. Endometrial cancer (Endo), breast cancer (BRCA), and lung squamous cell carcinoma (LUSC) are among the top five cancer types with PIK3CA alterations (cBioPortal for Cancer Genomics). In BRCA and Endo, PIK3CA is mutated in 35% and 50% of patients, respectively. While in LUSC, ∼40% of patients carry a somatic copy number alteration of PIK3CA amplification. Examining the RNA-protein correlation of PIK3CA in each of these three cancer types, divided into patients with and without PIK3CA alteration (*i.e.* mutation or copy number aberration), we found that in PIK3CA amplified tumors (in LUSC), the RNA-protein correlation is higher than in non-amplified tumors (0.76 vs. 0.45; Fig. 2*B*). Interestingly, the opposite is true for PIK3CA missense mutations in Endo (Fig. 2*C*), and to a lesser extent in BRCA (Fig. 2*D*), where the RNA-protein correlation is higher in WT compared to mutant tumors (0.68 vs. 0.51 for Endo, 0.33 vs. 0.29 for BRCA). In the case of ErbB2, the RNA-protein correlation across all BRCA tumors is 0.48. Remarkably, when evaluating ErbB2 in breast tumors separated to ErbB2-amplified and non-amplified, the RNA-protein correlation of amplified tumors is 0.86, while the RNA-protein correlation of non-amplified tumors is only 0.26 (Fig. 2*E*). Since both PIK3CA and ErbB2 alterations reflect major driver events in tumor development, these results suggest that high RNA-protein correlations are associated with tumor functionality in the case of gene amplification. The lower correlation in the case of PIK3CA mutations shows that RNA measurements do not reflect protein abundance, and further research is necessary to investigate whether protein levels are associated with drug response. While it has been shown that protein levels usually compensate for copy number alterations (13), this finding suggests that this compensation does not take place in the cases where the genomic event is critical for tumor development/progression.

### Impact of Protein–RNA Correlations on Cancer Classification

**Fig. 3:**
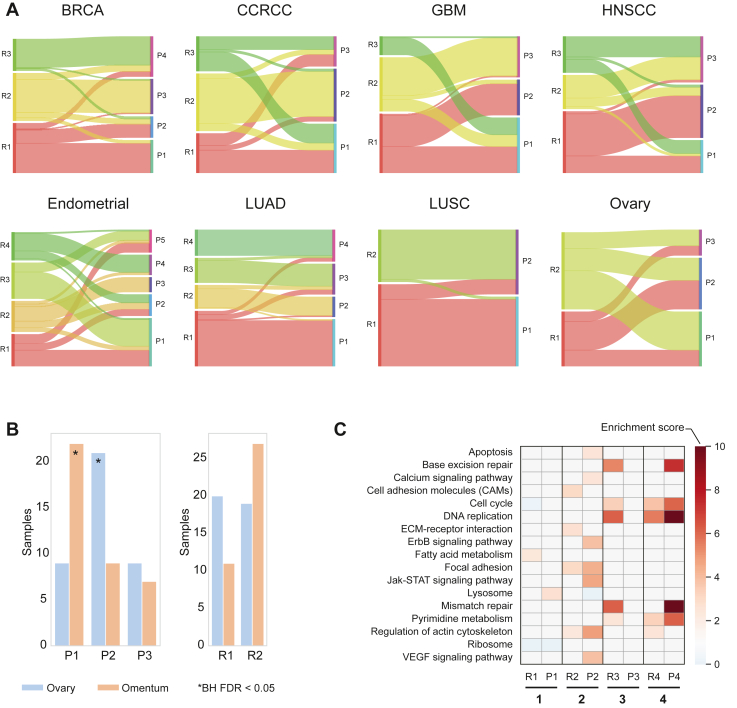
**Differential tumor classification using RNA or protein data.***A*, alluvial plots show tumor distribution among RNA or protein clusters. RNA-based clusters are marked as R1, R2, R3, etc., and proteomics-based clusters are marked as P1, P2, P3, etc. LUAD and LUSC have very similar classifications, while other cancer types present substantial differences among tumor subtypes. *B*, bar plot shows the enrichment of ovarian cancers in the ovaries and the omentum. These clinical features are significantly enriched in protein clusters 1 and 2. *C*, functional enrichment analysis of GO and KEGG pathways shows that despite the almost identical classification of LUAD based on protein or RNA, enriched processes markedly differ.

One of the goals of many OMICS studies is to classify tumors in an unbiased way, in order to identify potential prognostic and predictive markers as well as drug targets that may be applicable to large patient groups. Optimization of the numbers of clusters and the separation of tumor samples has been often performed using the consensus-clustering algorithm, which utilizes normalized transcriptomic or proteomic data to determine the most robust grouping of samples (43, 44). In our previous studies, we found substantial differences in RNA and protein-based classifications (19, 45); however, these may have been partially confounded by the tumor heterogeneity, as samples were not taken from the exact same specimen. Here, the analyses of the same samples overcome this limitation but still show marked differences in most tumor types (Fig. 3*A*). In agreement with the overall high protein-RNA correlation, classification of lung cancers (LUAD and LUSC) separated the tumors into four and two clusters, respectively, with almost perfect concordance between RNA and protein-based clusters. All other cancer types examined here show poorer agreement, having different numbers of clusters as well as mismatch of the samples. A comparison to the original CPTAC clustering shows better concordance with the protein-based clusters in three of the five cancer types that originally included clustering analyses (BRCA, GBM, and HNSCC), while the lung cancers showed high concordance between RNA and protein also in our analyses (Supplemental Table S3). Clustering of LUSC differed from the original integrated clustering, presumably due to the contribution of genomics and posttranslational modifications in this case. Deeper investigation of the tumors within each cluster, and analysis of the enriched processes that differentiate between clusters shows the functional impact of discordant classification. For example, ovarian tumor samples were separated into two clusters based on RNA and three clusters based on protein expression. Of note, each of the RNA-based clusters separated into each of the three protein-based clusters with very low concordance. Enrichment analysis of clinical annotations showed that the protein-based classification captures differences between tumors located in the ovaries (cluster 1) and tumors located in the omentum (cluster 2, Fig. 3*B*). Analysis of the proteins that significantly differentiate between the protein clusters shows that cluster 1 has high expression of matrix proteins and adhesion molecules, thus reflecting the fibrotic nature of the omentum (Supplemental Table S4). This separation also reflects a major clinical difference in the tumor response to chemotherapy. No such enrichment is seen in the RNA clusters.

To examine the functional differences between clusters, we performed differential expression tests between the clusters of each layer (RNA and protein) and extracted the significantly different proteins. For a comparable analysis, we selected the proteins and transcripts that were commonly found in each tumor type. Functional enrichment analysis of gene ontology (GO) annotations and KEGG pathways was performed to show cluster-specific functional characteristics at both the RNA and the protein layers. We were intrigued to find that even in tumor types such as LUAD, in which there was an almost perfect agreement in subtype separation, the functional enrichments were significantly different (Fig. 3*C* and Supplemental Table S5). As an example, cluster 2 showed many more enriched pathways at the protein level than the RNA level. Additionally, several pathways (*e.g.* base excision repair [BER] and mismatch repair [MMR]) were enriched in one cluster based on protein expression, and in another cluster based on RNA expression. Overall, these results hint towards the challenging functional interpretation of the proteogenomic classification, and raise the need for a deeper evaluation of prognostic implications once more data and follow-up information are collected.

## Conclusion

Our analyses show the major divergences between protein and RNA expression analyses in the context of translational cancer research and further highlight the challenge of predicting protein expression levels from RNA. Despite the exceptional contribution of CPTAC and the generation of large clinical proteogenomic datasets, critical gaps still need to be bridged in order to apply discoveries from tissue proteomic data in a clinical setting. First, most proteomic studies to date lack sufficient clinical information regarding survival and drug response, and these are critical for the elucidation of cancer mechanisms and identification of cancer vulnerabilities. Second, the addition of post-translational modifications (PTMs) may have important translational implications. CPTAC studies include PTM analyses (mostly phosphorylations and acetylations), and multiple algorithms have been developed in order to transform the complex PTM data into coherent pathways (46, 47, 48, 49, 50, 51). Nevertheless, further experimental and computational developments are required in order to transform these challenging data to valid predictions of treatment responses. Additionally, given the major challenge of internal tumor heterogeneity, combining the proteomic data with the spatial tumor characteristics would further increase our understanding of cancer dynamics and evolution and increase predictive accuracy. Finally, it is critical to bridge the technological gap and implement MS-based technologies in medical centers. Similar to implementation of genomic approaches, we envision that simple and robust MS analyses can be used in clinical settings and enable rapid analyses of tumors and body fluids. Altogether, we anticipate that proteomics-based cancer research will dramatically increase the impact of OMICS analyses, highlight novel therapeutic approaches, and increase the overall success of clinical trials.

## Experimental Procedures

### Data Collection

All the data used in this study were previously published by CPTAC. Both proteomic and transcriptomic data were obtained using the CPTAC python package (GitHub - PayneLab/cptac: Python packaging for CPTAC data) with CPTAC version 1.1.1. Specifically, we analyzed data from eight cancer types: breast cancer (29), ovarian cancer (36), clear cell renal cell carcinoma (34) (CCRCC), glioblastoma (27) (GBM), head and neck squamous cell carcinoma (30) (HNSCC), lung adenocarcinoma (31) (LUAD), lung squamous cell carcinoma (28) (LUSC) and endometrial cancer (35).

### Statistical Analysis

Protein values used for all analyses in this study are TMT ratios normalized to common reference mix channels as described in CPTAC publications. Transcript values used for correlation analysis were based on counts or FPKM values (depending on CPTAC python package availability) while all transcriptomics matrices were converted to FPKM values for classification analysis. For each protein-transcript pair, we calculated the Spearman rank correlation. The consensus clustering algorithm (43, 44) was used for the classification of both proteomic and transcriptomic data. Differential expression analysis was performed using *t* test with permutation-based false discovery rate (FDR) of 0.01. Enrichment analysis of either clinical annotations or processes and pathways was performed using Fisher Exact test with Benjamini-Hochberg FDR of 0.05. All statistical analysis were performed using R, Python and the Perseus computational platform (52).

## Supplemental data

This article contains supplemental data.

## Conflict of interest

G. A. is an employee and T. G. is an advisor of Protai Bio.

## References

[1] Martinez-Jimenez F., Muinos F., Sentis I., Deu-Pons J., Reyes-Salazar I., Arnedo-Pac C. (2020). A compendium of mutational cancer driver genes. Nat. Rev. Cancer.

[2] Cieslik M., Chinnaiyan A.M. (2018). Cancer transcriptome profiling at the juncture of clinical translation. Nat. Rev. Genet..

[3] Mani D.R., Krug K., Zhang B., Satpathy S., Clauser K.R., Ding L. (2022). Cancer proteogenomics: current impact and future prospects. Nat. Rev. Cancer.

[4] Macklin A., Khan S., Kislinger T. (2020). Recent advances in mass spectrometry based clinical proteomics: applications to cancer research. Clin. Proteomics.

[5] Doll S., Gnad F., Mann M. (2019). The case for proteomics and phospho-proteomics in personalized cancer medicine. Proteomics Clin. Appl..

[6] Wu P., Heins Z.J., Muller J.T., Katsnelson L., de Bruijn I., Abeshouse A.A. (2019). Integration and analysis of CPTAC proteomics data in the context of cancer genomics in the cBioPortal. Mol. Cell Proteomics.

[7] Nagaraj N., Wisniewski J.R., Geiger T., Cox J., Kircher M., Kelso J. (2011). Deep proteome and transcriptome mapping of a human cancer cell line. Mol. Syst. Biol..

[8] Schwanhausser B., Busse D., Li N., Dittmar G., Schuchhardt J., Wolf J. (2011). Global quantification of mammalian gene expression control. Nature.

[9] Lundberg E., Fagerberg L., Klevebring D., Matic I., Geiger T., Cox J. (2010). Defining the transcriptome and proteome in three functionally different human cell lines. Mol. Syst. Biol..

[10] Jovanovic M., Rooney M.S., Mertins P., Przybylski D., Chevrier N., Satija R. (2015). Immunogenetics. Dynamic profiling of the protein life cycle in response to pathogens. Science.

[11] Cheng Z., Teo G., Krueger S., Rock T.M., Koh H.W., Choi H. (2016). Differential dynamics of the mammalian mRNA and protein expression response to misfolding stress. Mol. Syst. Biol..

[12] Upadhya S.R., Ryan C.J. (2022). Experimental reproducibility limits the correlation between mRNA and protein abundances in tumor proteomic profiles. Cell Rep. Met..

[13] Cheng P., Zhao X., Katsnelson L., Camacho-Hernandez E.M., Mermerian A., Mays J.C. (2022). Proteogenomic analysis of cancer aneuploidy and normal tissues reveals divergent modes of gene regulation across cellular pathways. Elife.

[14] Buccitelli C., Selbach M. (2020). mRNAs, proteins and the emerging principles of gene expression control. Nat. Rev. Genet..

[15] Schukken K.M., Sheltzer J.M. (2022). Extensive protein dosage compensation in aneuploid human cancers. Genome Res..

[16] Stingele S., Stoehr G., Peplowska K., Cox J., Mann M., Storchova Z. (2012). Global analysis of genome, transcriptome and proteome reveals the response to aneuploidy in human cells. Mol. Syst. Biol..

[17] Geiger T., Cox J., Mann M. (2010). Proteomic changes resulting from gene copy number variations in cancer cells. PLoS Genet..

[18] Shenoy A., Belugali Nataraj N., Perry G., Loayza Puch F., Nagel R., Marin I. (2020). Proteomic patterns associated with response to breast cancer neoadjuvant treatment. Mol. Syst. Biol..

[19] Yanovich G., Agmon H., Harel M., Sonnenblick A., Peretz T., Geiger T. (2018). Clinical proteomics of breast cancer reveals a novel layer of breast cancer classification. Cancer Res..

[20] Leo I.R., Aswad L., Stahl M., Kunold E., Post F., Erkers T. (2022). Integrative multi-omics and drug response profiling of childhood acute lymphoblastic leukemia cell lines. Nat. Commun..

[21] Eckert M.A., Coscia F., Chryplewicz A., Chang J.W., Hernandez K.M., Pan S. (2019). Proteomics reveals NNMT as a master metabolic regulator of cancer-associated fibroblasts. Nature.

[22] Coscia F., Lengyel E., Duraiswamy J., Ashcroft B., Bassani-Sternberg M., Wierer M. (2018). Multi-level proteomics identifies CT45 as a chemosensitivity mediator and immunotherapy target in ovarian cancer. Cell.

[23] Mertins P., Mani D.R., Ruggles K.V., Gillette M.A., Clauser K.R., Wang P. (2016). Proteogenomics connects somatic mutations to signalling in breast cancer. Nature.

[24] Zhang H., Liu T., Zhang Z., Payne S.H., Zhang B., McDermott J.E. (2016). Integrated proteogenomic characterization of human high-grade serous ovarian cancer. Cell.

[25] Zhang B., Wang J., Wang X., Zhu J., Liu Q., Shi Z. (2014). Proteogenomic characterization of human colon and rectal cancer. Nature.

[26] Woo S., Cha S.W., Bonissone S., Na S., Tabb D.L., Pevzner P.A. (2015). Advanced proteogenomic analysis reveals multiple peptide mutations and complex immunoglobulin peptides in colon cancer. J. Proteome Res..

[27] Wang L.B., Karpova A., Gritsenko M.A., Kyle J.E., Cao S., Li Y. (2021). Proteogenomic and metabolomic characterization of human glioblastoma. Cancer Cell.

[28] Satpathy S., Krug K., Jean Beltran P.M., Savage S.R., Petralia F., Kumar-Sinha C. (2021). A proteogenomic portrait of lung squamous cell carcinoma. Cell.

[29] Krug K., Jaehnig E.J., Satpathy S., Blumenberg L., Karpova A., Anurag M. (2020). Proteogenomic landscape of breast cancer tumorigenesis and targeted therapy. Cell.

[30] Huang C., Chen L., Savage S.R., Eguez R.V., Dou Y., Li Y. (2021). Proteogenomic insights into the biology and treatment of HPV-negative head and neck squamous cell carcinoma. Cancer Cell.

[31] Gillette M.A., Satpathy S., Cao S., Dhanasekaran S.M., Vasaikar S.V., Krug K. (2020). Proteogenomic characterization reveals therapeutic vulnerabilities in lung adenocarcinoma. Cell.

[32] Clark D.J., Dhanasekaran S.M., Petralia F., Pan J., Song X., Hu Y. (2019). Integrated proteogenomic characterization of clear cell renal cell carcinoma. Cell.

[33] Cao L., Huang C., Cui Zhou D., Hu Y., Lih T.M., Savage S.R. (2021). Proteogenomic characterization of pancreatic ductal adenocarcinoma. Cell.

[34] Clark D.J., Dhanasekaran S.M., Petralia F., Pan J., Song X., Hu Y. (2020). Integrated proteogenomic characterization of clear cell renal cell carcinoma. Cell.

[35] Dou Y., Kawaler E.A., Cui Zhou D., Gritsenko M.A., Huang C., Blumenberg L. (2020). Proteogenomic characterization of endometrial carcinoma. Cell.

[36] McDermott J.E., Arshad O.A., Petyuk V.A., Fu Y., Gritsenko M.A., Clauss T.R. (2020). Proteogenomic characterization of ovarian HGSC implicates mitotic kinases, replication stress in observed chromosomal instability. Cell Rep. Med..

[37] Vasaikar S., Huang C., Wang X., Petyuk V.A., Savage S.R., Wen B. (2019). Proteogenomic analysis of human colon cancer reveals new therapeutic opportunities. Cell.

[38] Boisvert F.M., Ahmad Y., Gierlinski M., Charriere F., Lamont D., Scott M. (2012). A quantitative spatial proteomics analysis of proteome turnover in human cells. Mol. Cell Proteomics.

[39] Dorrbaum A.R., Kochen L., Langer J.D., Schuman E.M. (2018). Local and global influences on protein turnover in neurons and glia. Elife.

[40] Sanchez-Vega F., Mina M., Armenia J., Chatila W.K., Luna A., La K.C. (2018). Oncogenic signaling pathways in the cancer genome atlas. Cell.

[41] Clevers H., Nusse R. (2012). Wnt/beta-catenin signaling and disease. Cell.

[42] Alon U. (2007). Network motifs: theory and experimental approaches. Nat. Rev. Genet..

[43] Wilkerson M.D., Hayes D.N. (2010). ConsensusClusterPlus: a class discovery tool with confidence assessments and item tracking. Bioinformatics.

[44] Monti S., Tamayo P., Mesirov J., Golub T. (2003). Consensus clustering: a resampling-based method for class discovery and visualization of gene expression microarray data. Machine Learn..

[45] Yanovich-Arad G., Ofek P., Yeini E., Mardamshina M., Danilevsky A., Shomron N. (2021). Proteogenomics of glioblastoma associates molecular patterns with survival. Cell Rep..

[46] Beekhof R., van Alphen C., Henneman A.A., Knol J.C., Pham T.V., Rolfs F. (2019). INKA, an integrative data analysis pipeline for phosphoproteomic inference of active kinases. Mol. Syst. Biol..

[47] Yilmaz S., Ayati M., Schlatzer D., Cicek A.E., Chance M.R., Koyuturk M. (2021). Robust inference of kinase activity using functional networks. Nat. Commun..

[48] Rudolph J.D., de Graauw M., van de Water B., Geiger T., Sharan R. (2016). Elucidation of signaling pathways from large-scale phosphoproteomic data using protein interaction networks. Cell Syst..

[49] Piersma S.R., Valles-Marti A., Rolfs F., Pham T.V., Henneman A.A., Jimenez C.R. (2022). Inferring kinase activity from phosphoproteomic data: tool comparison and recent applications. Mass Spectrom Rev..

[50] Casado P., Rodriguez-Prados J.C., Cosulich S.C., Guichard S., Vanhaesebroeck B., Joel S. (2013). Kinase-substrate enrichment analysis provides insights into the heterogeneity of signaling pathway activation in leukemia cells. Sci. Signal..

[51] Crowl S., Jordan B.T., Ahmed H., Ma C.X., Naegle K.M. (2022). KSTAR: an algorithm to predict patient-specific kinase activities from phosphoproteomic data. Nat. Commun..

[52] Tyanova S., Temu T., Sinitcyn P., Carlson A., Hein M.Y., Geiger T. (2016). The Perseus computational platform for comprehensive analysis of (prote)omics data. Nat. Met..

