# Supplementary material for: Functional Impact of Protein–RNA Variation in Clinical Cancer Analyses
Source: Mol Cell Proteomics. 2023 Jun 7;22(7):100587. doi: 10.1016/j.mcpro.2023.100587 (PMC10388586; doi:10.1016/j.mcpro.2023.100587)
Supplement: Supplementary Table S1 [file mmc1.docx]

**Supplementary Table 1:** Number of samples and protein IDs in each analysis in the study

|  | **num samples** | **num genes used for rna-prot correlations (Figure 1)** | **number of drug targets per cancer type**  **(Figure 2)** | **number of samples used for clustering-rna**  **(Figure 3)** | **number of samples used for clustering-prot**  **(Figure 3)** |
| --- | --- | --- | --- | --- | --- |
| **BRCA** | 122 | 5870 | 89 | 122 | 122 |
| **CCRCC** | 110 | 6619 | 102 | 110 | 110 |
| **Endo** | 95 | 8165 | 118 | 95 | 95 |
| **HNSCC** | 109 | 7648 | 114 | 109 | 109 |
| **LUAD** | 110 | 7040 | 103 | 110 | 110 |
| **LUSC** | 108 | 8035 | 120 | 108 | 108 |
| **Ovary** | 82 | 6439 | 107 | 82 | 83 |
| **GBM** | 99 | 8451 | 117 | 99 | 99 |
